# Association between COVID-19 vaccination and progression to severe outcomes in hospitalized COVID-19 patients in Hungary during the pre-Omicron era of the COVID-19 pandemic

**DOI:** 10.1016/j.virusres.2025.199633

**Published:** 2025-09-18

**Authors:** Zoltán Vokó, Gergő Túri, Beatrix Oroszi, Éva Belicza, Tamás Kováts, Veronika Müller, János Réthelyi, Attila Szijártó, Tamás Masszi, István Takács, János Gál, Zsolt Göböl, Attila Szabó, Csaba Varga, Dávid Becker, Béla Merkely

**Affiliations:** a Center for Health Technology Assessment, Semmelweis University, Üllői út 25 1091 Budapest, Hungary; b Center for Pharmacology and Drug Research & Development, Semmelweis University, Üllői út 26 1085 Budapest, Hungary; c Syreon Research Institute, Mexikói út 65/A 1142 Budapest, Hungary; d National Laboratory for Health Security, Epidemiology and Surveillance Centre Semmelweis University, Üllői út 25 1091 Budapest, Hungary; e Doctoral School of Health Sciences, University of Debrecen, Nagyerdei krt. 94 4032 Debrecen, Hungary; f Data-Driven Health Division of National Laboratory for Health Security, Health Services Management Training Centre, Semmelweis University, Kútvölgyi út 2 1125 Budapest, Hungary; g Department of Pulmonology, Semmelweis University, Tömő u. 25-29 1083 Budapest, Hungary; h Department of Psychiatry and Psychotherapy, Semmelweis University, Balassa u. 6 1083 Budapest, Hungary; i Department of Surgery, Transplantation and Gastroenterology, Semmelweis University, Üllői út 78 1082 Budapest, Hungary; j Department of Internal Medicine and Haematology, Semmelweis University, Szentkirályi u. 46 1088, Budapest, Hungary; k Department of Anesthesiology and Intensive Therapy, Semmelweis University, Üllői út 78/B, 1082, Budapest, Hungary; l Multidisciplinary Day Surgery Clinic, Semmelweis University, Gyulai Pál u. 2 1085 Budapest, Hungary; m Pediatric Center, Semmelweis University, Budapest, Hungary; n Department of Emergency Medicine, Semmelweis University, Bókay János u. 53-54 1083 Budapest, Hungary; o Heart and Vascular Center of Semmelweis University, Városmajor utca 68 1122 Budapest, Hungary

**Keywords:** COVID-19, Vaccination, SARS-CoV-2, Clinical outcomes, Severe outcome, Prognosis, Hospitalized patients, Hungary

## Abstract

COVID-19 vaccines reduce hospitalization risk, but data on severe outcomes are limited. We analyzed the impact of COVID-19 vaccination on severe outcomes in hospitalized patients in Hungary during the pre-Omicron era, addressing a regional knowledge gap. This retrospective study included hospitalized patients with PCR-confirmed COVID-19 (March 2020 – December 2021) who were categorized as unvaccinated, primary immunized, or booster-vaccinated. Outcomes included oxygen therapy, ventilation types, ECMO, and death, with the most severe outcome as the primary outcome and individual outcomes as secondary measures. Polytomous logistic regression calculated relative risk ratios for the primary outcome and COVID-19 vaccination status, while logistic regression estimated odds ratios for individual outcomes. During the study, 7575 patients were hospitalized with PCR-confirmed COVID-19: 6420 (84.8 %) were unvaccinated, 1016 (13.4 %) received a primary vaccination series, and 139 (1.8 %) had received a booster dose. COVID-19 vaccination reduced the risk of both invasive ventilation and in-hospital death as the most severe outcome by 50 % within 12 months (relative risk ratio [RRR]: 0.52, 95 % CI: 0.30–0.89; 0.50, 95 % CI: 0.41–0.61). Booster doses within six months decreased the risk of in-hospital death to a similar extent (RRR 0.46, 95 % CI: 0.30–0.72). Primary and booster vaccination reduced the risk of progression to severe outcomes in hospitalized COVID-19 patients.

## Highlights

• Vaccination reduced the risk of severe outcomes in hospitalized COVID-19 patients.
• The risk of invasive ventilation and in-hospital death was reduced by 50 %.
• The effect of primary and booster-vaccination did not differ substantially.

## 1 Introduction

Several real-world studies, such as test-negative case-control studies, have assessed the effectiveness of COVID-19 vaccines in preventing symptomatic illness and hospitalization. These studies have demonstrated that COVID-19 vaccines are effective in reducing the risk of hospitalization, although the duration of the protective effect is not yet well understood (Antunes et al., 2024a, 2024b; Lanièce Delaunay et al., 2024). Test-negative design poses more challenges for evaluating vaccine effectiveness against severe outcomes including differences in hospitalization thresholds by vaccination status, misclassification from reduced test sensitivity later in illness, and severe outcomes in hospitalized, test-negative controls may be driven by other pathogens or chronic diseases that are unequally distributed by vaccination status. (Shi et al., 2023). Furthermore, in the era of the Omicron variant, an increasing number of factors may cause bias when assessing the effectiveness of COVID-19 vaccination against mild and particularly against severe outcomes (Doll et al., 2022; Maurel et al., 2024; Qasmieh et al., 2022; Shi et al., 2023).

The existing literature on the relationship between vaccination status and the progression to severe COVID-19 outcomes is limited, particularly in Central and Eastern European countries where vaccination portfolios are diverse. Most studies in this field originate from North America and Western Europe (Lin et al., 2022; Van Diepen et al., 2023; Whittaker et al., 2022). Hungary, during the first four pandemic waves (2020–2021), saw 1.25 million cases and over 39,000 deaths (European Centre for Disease Prevention and Control 2023). Vaccines became available after the second wave, significantly impacting subsequent waves. Significant excess deaths were identified for several weeks around the peak of the second, third, and fourth pandemic waves (Oroszi et al., 2022, 2021). By the end of 2021, 67.7 % of individuals aged 12 and older had received primary immunization, and 36 % had received boosters. A unique element of the Hungarian vaccination program was the high proportion of heterologous vaccination, whereby the primary vaccination was carried out with a range of vaccines (European Centre for Disease Prevention and Control 2023; Oroszi et al., 2022). In contrast, the booster vaccination was predominantly administered using mRNA-based vaccines. Despite the extensive range of vaccines available in Hungary, primary and booster dose vaccination coverage fell below the EU average (European Centre for Disease Prevention and Control 2023), partly due to declining public confidence in vaccine effectiveness. This was due to a reduction in the perceived risk of COVID-19 and increased skepticism regarding the vaccine's ability to provide protection (Ligeti et al., 2024). Evidence demonstrating vaccination’s role in preventing severe disease progression is therefore crucial to rebuilding confidence and improving uptake.

Semmelweis University, which managed 18 % of Central Hungary’s cases, played a vital role during the pandemic, treating large numbers of COVID-19 patients, including those with complex needs.

To address gaps in existing knowledge, we aimed to evaluate the association between COVID-19 vaccination and the progression to severe outcomes among hospitalized patients in Hungary during the pre-Omicron era, given the limited availability of data for this region and period.

## 2 Methods

### 2.1 Study design, setting and population

This retrospective study involved inpatients at Semmelweis University who received treatment for COVID-19 and had SARS-CoV-2 infection confirmed by polymerase chain reaction (PCR) testing, from the beginning of the pandemic (March 2020) until December 31, 2021.

Patients were identified retrospectively based on ICD-10 code (using the U0710). The association between COVID-19 vaccination status at admission and in-hospital outcomes was investigated. Patients were categorized into three groups based on their vaccination status at admission: a) unvaccinated, b) those who had completed primary immunization at least 14 days before admission, and c) those who had received a booster dose at least 14 days prior. Six different SARS-CoV-2 vaccines were administered in Hungary: AZD1222 (AstraZeneca), Ad26.COV2.S (Janssen), BNT162b2 (Pfizer-BioNTech), HB02 (Sinopharm), Gam-COVID-Vac (Sputnik V), and mRNA-1273 (Moderna). Primary immunization consisted of two doses, except for the Janssen vaccine. We excluded 350 patients who had received only the first dose of the primary series or completed the second one within two weeks before admission.

Data on medical history were retrieved from the National Health Insurance Fund's inpatient and outpatient databases, using ICD-10 codes to identify comorbidities. (Supplementary Document 1). Patient demographics and comorbidities — such as cardiovascular diseases (including ischemic heart disease and heart failure), cerebrovascular diseases, type 2 diabetes mellitus, chronic obstructive pulmonary disease (COPD), and malignancies — were collected as covariates of interest. A condition was classified as part of the medical history if patients had received care for it between January 1, 2015, and the date of their COVID-19 hospitalization.

### 2.2 Clinical outcomes

The primary outcomes was defined as the most severe event experienced by the patient during hospitalization, including oxygen therapy, non-invasive ventilation (intermittent positive pressure ventilation through specialized masks, excluding simple continuous positive airway pressure [CPAP]), invasive ventilation (intermittent positive pressure mechanical ventilation with endotracheal intubation, intermittent positive pressure mechanical ventilation with JET technique, or nasal CPAP in children), extracorporeal membrane oxygenation (ECMO), and in-hospital mortality. Outcome data were sourced from the Semmelweis University hospital information system. Each patient was classified based on the most severe outcome they experienced. The individual components of the primary outcome were prespecified as key separate secondary outcomes. Patients were followed up until discharge for the same hospitalization or until in-hospital death.

### 2.3 Statistical analysis

Descriptive statistics were employed to examine the distribution of baseline variables. Continuous variables were presented as means with standard deviations (SD), while categorical variables were expressed as counts and percentages. Polytomous logistic regression was employed to analyse the association between the most severe outcome and COVID-19 vaccination status at admission, adjusted for sex, age, and comorbidities including cardiovascular diseases (ischemic heart disease and heart failure), cerebrovascular diseases, type 2 diabetes mellitus, COPD, and malignancies. Relative risk ratios were estimated as effect measures. As for the key secondary outcomes, logistic regression models were fitted with the different outcomes as independent variables and using the same covariates as in the polytomous logistic regression analysis. The effect of vaccination status was adjusted for sex, age, and comorbidities. Odds ratios were estimated as effect measures. Analyses were performed with the software package STATA 18.0.

## 3 Results

A total of 7575 patients were involved in the study, of which 6420 (84.8 %) were not vaccinated, 1016 (13.4 %) had completed primary vaccination, and 139 (1.8 %) received booster vaccination. They were hospitalized for an average of 12.3 (SD: 20.7) days, median 9 days. Table 1 describes the baseline characteristics of patients. Most of the participants were adults; only 216 (2.85 %) children under 18 years were involved, of whom only one was vaccinated. The proportion of males was about the same among the unvaccinated and vaccinated, with a slightly higher proportion of males among those with booster vaccination. The unvaccinated patients were younger, with a lower prevalence of comorbidities than the vaccinated and booster-vaccinated patients.

**Table 1:** Patient characteristics at baseline.

	Vaccination status		
Characteristics	Not vaccinated (N=6420)	Completed primary COVID-19 vaccination (N=1016)	Booster-vaccinated (N=139)
Sex – N( %)			
Male	3285 (51)	500 (49)	78 (56)
Female	3135 (49)	516 (51)	61 (44)
Age (year) (mean (SD))	58. 5 (20.1)	63. 1 (17.6)	72. 1 (15.2)
Comorbidites – N( %)			
Cardiovascular diseases	1799 (28.0)	356 (35.0)	64 (46.0)
Cerebrovascular diseases	1188 (18.5)	245 (24.1)	37 (26.6)
Type-2 diabetes mellitus	1382 (21.5)	332 (32.7)	48 (34.5)
COPD	1217 (19.0)	208 (20.5)	43 (30.9)
Malignancy	903 (14.1)	194 (19.1)	41 (29.5)

SD: Standard deviation, COPD: chronic obstructive pulmonary disease

Most of the vaccinated patients received Pfizer or Sinopharm vaccines as primary vaccination: 467 patients received Pfizer (40.4 %), 341 Sinopharm (29.5 %), 155 Astra (13.4 %), 124 Sputnik (10.7 %), 62 Moderna (5.4 %), and 6 patients Janssen vaccine (0.52 %).

A total of 4256 (56.2 %) reached either component of the composite primary outcome during their hospital stay (Table 2). The occurrence of the most severe outcomes per patient by vaccination status is described in Table 2, where all patients were categorized into the worst outcome experienced during follow-up. The proportion of patients who did not even require oxygen therapy (i.e., did not experience any of the outcomes of the study) was similar in the three groups (45.4 %, 47.0 % and 43.9 %). Few patients received non-invasive or invasive ventilation therapy as the most severe outcome. Around 30 % of patients did not have a more severe outcome than oxygen therapy. The raw proportion of in-hospital death was lowest among patients who completed primary vaccination (17.4 %), and the highest among booster vaccinated patients (26.6 %).

**Table 2:** Occurrence of the most severe primary outcomes by COVID-19 vaccination status.

Outcome	Not vaccinated (N=6420)	Completed primary COVID-19 vaccination (N=1016)	Booster-vaccinated (N=139)
Oxygen therapy – no ( %)	1913 (29.8)	321 (31.6)	36 (25.9)
Non-invasive ventilation – no ( %)	152 (2.4)	25 (2.5)	2 (1.4)
Invasive ventilation – no ( %)	160 (2.5)	15 (1.5)	3 (2.2)
ECMO – no ( %)	11 (0.17)	0 (0.0)	0 (0.0)
Death – no ( %)	1404 (21.9)	177 (17.4)	37 (26.6)

ECMO: Extracorporeal Membrane Oxygenation

Table 3 shows the prevalence of key secondary outcomes by COVID-19 vaccination status. The proportion of those with complete primary and booster vaccinations who received oxygen therapy and non-invasive ventilation was similar to that of the unvaccinated. However, a lower proportion of primary vaccinated and booster-vaccinated patients received invasive ventilation support and ECMO.

**Table 3:** Occurrence of each separate outcomes by COVID-19 vaccination status.

Outcome	Not vaccinated (N=6420)	Completed primary COVID-19 vaccination (N=1016)	Booster-vaccinated (N=139)
Oxygen therapy – no ( %)	2798 (43.6)	463 (45.6)	61 (43.9)
Non-invasive ventilation – no ( %)	356 (5.6)	57 (5.6)	6 (4.3)
Invasive ventilation – no ( %)	517 (8.0)	45 (4.4)	3 (2.2)
ECMO – no ( %)	44 (0.69)	3 (0.30)	0 (0.0)
Death – no ( %)	1404 (21.9)	177 (17.4)	37 (26.6)

ECMO: Extracorporeal Membrane Oxygenation

Vaccination within 12 months before hospitalization reduced the risks for almost all severe outcomes (Table 4). Vaccination significantly reduced the risks of invasive ventilation by an odds ratio of 0.52 (95 % CI: 0.38-0.71) and in-hospital death by an odds ratio of 0.55 (95 % CI: 0.46-0.67). Booster vaccination within six months reduced the risks of death to a similar extent and further reduced the risk of all other outcomes. No significant risk reduction was observed for the oxygen therapy and non-invasive ventilation outcomes.

**Table 4:** Association between COVID-19 vaccination status and risk of each different outcomes corrected for age, sex, history of type-2 diabetes, cardiovascular diseases, COPD and malignancies as estimated from separate logistic regression models.

Outcome	Not vaccinated	Completed Primary COVID-19 Vaccination	Booster-vaccinated
		Odds ratio and 95 % CI	
Oxygen therapy	reference	1.00 (0.87; 1.14)	0.76 (0.54; 1.08)
Non-invasive ventilation		1.00 (0.74; 1.33)	0.72 (0.31;1.64)
Invasive ventilation		0.52 (0.38; 0.71)	0.24 (0.08; 0.76)
ECMO		0.54 (0.17; 1.76)	-
Death		0.55 (0.46; 0.67)	0.61 (0.41; 0.92)

95 % CI: 95 % confidence interval, ECMO: Extracorporeal Membrane Oxygenation

When each patient was classified into the worst outcome they experienced, we found that vaccination within 12 months before hospitalization reduced the risks of all outcomes (Table 5). Vaccination significantly reduced the risk of invasive ventilation with a relative risk ratio (RRR) of 0.52 (95 % CI: 0.30-0.89) and death (RRR: 0.50, 95 % CI: 0.41-0.61) within 12 months compared to no vaccination. The effect estimates of booster vaccination were in line with these, but they were very imprecise in the case of the rare outcomes, however large and significant for oxygen therapy and death.

**Table 5:** Association between experiencing more severe outcome* and COVID-19 vaccination status corrected for age, sex, history of type-2 diabetes mellitus, cardiovascular diseases, COPD and malignancies as estimated from a polytomous logistic regression model using the non-vaccinated patient group as reference category.

	Relative risk ratio† (95 % CI)	
Outcome	Completed primary COVID-19 vaccination	Booster-vaccinated
Oxygen therapy	0.84 (0.71; 0.98)	0.57 (0.37; 0.88)
Non-invasive ventilation	0.79 (0.56; 1.35)	0.48 (0.12; 2.01)
Invasive ventilation	0.52 (0.30; 0.89)	0.70 (0.21; 2.27)
Death	0.50 (0.41; 0.61)	0.46 (0.30; 0.72)

* We classified each patient into the category of the worst outcome they experienced.

† Compared to non-vaccinated persons. E.g., the relative risk ratio of death of 0.50 in vaccinated patients means the ratio of the relative risk of death compared to the risk of no ventilation support (best outcome) among vaccinated divided with the same relative risk among non-vaccinated patients.

95 % CI: 95 % confidence interval

If all patients were booster vaccinated, 56 % of them would not have experienced any of the study outcomes, whereas only 42 % if none of them were vaccinated (Table 6). The main health benefit of primary and booster vaccination could be observed in the reduction of the probability of in-hospital death.

**Table 6:** Estimated marginal probabilities of the most severe outcome* from the multivariate logistic regression by COVID-19 vaccination status corrected for age, sex, history of type-2 diabetes mellitus, cardiovascular diseases, COPD and malignancies.

	Estimated marginal probability (95 % CI)		
Outcome	Not vaccinated	Completed primary COVID-19 vaccination	Booster-vaccinated
No ventilation support	0.42 (0.41; 0.43)	0.50 (0.47; 0.53)	0.56 (0.48; 0.64)
Oxygen therapy	0.30 (0.29; 0.31)	0.31 (0.28; 0.34)	0.24 (0.17; 0.31)
Non-invasive ventilation	0.023 (0.020; 0.027)	0.025 (0.015; 0.034)	0.016 (0.0; 0.037)
Invasive ventilation	0.024 (0.021; 0.030)	0.015 (0.008; 0,023)	0.024 (0.0; 0.050)
ECMO	0.002 (0.0007; 0.003)	-	-
Death	0.23 (0.22; 0.24)	0.16 (0.13; 0.17)	0.16 (0.11; 0.21)

* We classified each patient into the category of the worst outcome they experienced, and calculated the probabilities of each outcome in the total study population conditional on vaccination status.

95 % CI: 95 % confidence interval, ECMO: Extracorporeal Membrane Oxygenation

## 4 Discussion

The findings of our study indicate that COVID-19 vaccination was an effective intervention for reducing the risk of invasive ventilation and mortality. It decreased the risk of these severe outcomes by 50 % among patients hospitalized with the disease within 12 months of the vaccine administration, compared to no vaccination, in the pre-Omicron era (March 2020 – December 2021) in Hungary. During this period the successive dominant SARS-CoV-2 variants were, first the Wuhan strain, then the Alpha variant, and later the Delta variant. Furthermore, our results demonstrate that a booster vaccination administered within six months before hospitalization had a similar effect among patients who have been laboratory confirmed as having the disease and who need hospital care compared to those with no vaccination. The largest health benefit of vaccination among COVID-19 patients was due to the reduction of in-hospital mortality. We must also highlight that our results suggest that the risk of ECMO therapy was considerably reduced among vaccinated patients, too. However, the statistical evidence is weak due to the limited number of patients receiving such care.

While a substantial body of epidemiological research has been dedicated to assessing whether vaccination against COVID-19 reduces the risk of hospitalization due to the disease, this study aims to contribute to the existing literature by investigating the potential of vaccination to prevent the progression to more severe outcomes in patients hospitalized with SARS-CoV-2 infection.

The characteristics of our study groups are consistent with those of other studies that have used similar comparison groups. Several retrospective cohort studies covered the pre-Omicron period, when first the Wuhan strain, then the Alpha variant, and later the Delta variant was dominant, and provided separate data on the study population. As in our study, these studies found, that fully vaccinated and booster-vaccinated patients were older than unvaccinated patients (Cai et al., 2024; Mielke et al., 2022; Van Diepen et al., 2023). Some studies also found a higher proportion of people with chronic diseases among the fully vaccinated and those who had received a booster than among the unvaccinated (Mielke et al., 2022; Suzuki et al., 2024; Van Diepen et al., 2023).

Furthermore, other studies have demonstrated a comparable reduction in the relative risk of invasive ventilation, which aligns with our findings. A study conducted in the United States between August 2021 and January 2022, when the Delta variant was dominant, demonstrated that hospitalized individuals who had received the primary series of COVID-19 vaccination had a significantly reduced risk of requiring mechanical ventilation compared to unvaccinated individuals (Mielke et al., 2022). Additionally, a further reduction in the risks of serious complications associated with SARS-CoV-2 infection was observed among individuals who received a booster vaccine compared to unvaccinated individuals. A Canadian retrospective cohort study conducted between January 2021 and July 2022, during the Alpha, Delta, and Omicron dominant periods, also revealed that the median duration of mechanical ventilation was longer among the unvaccinated individuals than those who received the full primary vaccination against COVID-19, and these results were consistent across the three different variants (Van Diepen et al., 2023). A Norwegian study, conducted in the pre-Omicron era, from February 2021 to November 2021, demonstrated that individuals who received the full primary vaccination regimen exhibited significantly shorter hospital length of stay and lower ICU admissions rates than unvaccinated individuals (Whittaker et al., 2022). These findings indicate that the progression towards a severe illness can be prevented by vaccination against SARS-CoV-2.

However, the existing literature was not consistent regarding hospital mortality outcomes. Our study's results contribute to the evidence base indicating that primary vaccination significantly reduces the risk of in-hospital death. A multicenter cohort study from the United States of America conducted in the pre-Omicron era from August 2021 to November 2021 also demonstrated that hospitalized individuals who had received the primary series of COVID-19 vaccination had a significantly reduced risk of in-hospital mortality compared to unvaccinated individuals, to a similar extent as in our study (Mielke et al., 2022). A further reduction was observed among individuals who received a booster vaccine compared to unvaccinated individuals. Another study from the United States, published in 2023, conducted between the Alpha and Delta variant dominant period of January 2021 to December 2021, revealed a significant reduction in the length of hospital stay and the risk of mortality due to complications of SARS-CoV-2 infection in hospitalized patients who had received the full primary vaccination series against COVID-19 compared to those who had not been vaccinated (Lee et al., 2023). However, in some studies, also covering the pre-Omicron periods, no significant difference in in-hospital mortality was observed between the vaccinated and unvaccinated individuals (Van Diepen et al., 2023; Whittaker et al., 2022).

Our study had some limitations. The study period ended with week 52 of 2021, thereby encompassing the period preceding the emergence of the Omicron variant in Hungary, which commenced with week 1 of 2022. The Omicron variant presents several methodological challenges in measuring vaccine effectiveness, given its distinctive characteristics and the reduced incidence of severe disease it causes. Nevertheless, the protective effect against progression to a more severe disease course, provided by vaccination, may also apply when the Omicron variant is dominant. However, the evidence to support this needs to be more conclusive. For example, a study conducted in the USA during the Omicron period in 2022-2023 revealed that individuals who had received an updated bivalent vaccination against SARS-CoV-2 exhibited a significantly reduced risk of hospitalization and a lower risk of experiencing a severe outcome, such as mechanical ventilation and ICU-level of care when hospitalized, in comparison to those who had not been vaccinated (Mielke et al., 2024). A Greek study, conducted between November 2022 to May 2023, when the Omicron-variant were dominant also found that receiving three or more doses of the COVID-19 vaccine offered substantial protection against various adverse outcomes (admission to intensive care unit, invasive mechanical ventilation, death) in hospitalized patients (Maltezou et al., 2024). A Japanese study conducted between July 2021 and August 2022 demonstrated that full primary vaccination reduced disease severity during the Delta- and Omicron-dominant phases. However, booster vaccination did not further enhance the protective effects against disease progression during the Omicron-dominant phase compared to full vaccination (Suzuki et al., 2024). A further limitation of this study is the relatively small sample size to measure very rare outcomes, especially among those vaccinated. Given that the booster vaccination campaign commenced only five months before the conclusion of our study, only a small proportion of patients who had received a booster vaccination were included in our analysis. Consequently, the sample size was insufficient to estimate some rare outcomes, such as ECMO treatment, among those who had received a booster vaccination.

## 5 Conclusions

The results of our study indicate that administering the full primary series of the SARS-CoV-2 vaccines, including diverse types commonly used in Eastern Europe, and booster doses were effective interventions in preventing the progression to severe outcomes in hospitalized patients with confirmed SARS-CoV-2 infection. These findings highlight the importance of different vaccine types against SARS-CoV-2 in mitigating severe disease and reducing the burden on healthcare systems during the most severe pandemic waves in Hungary.

## Funding

The research project (project number: 2020-1.1.6-JÖVŐ-2021-00013) was funded by the Ministry of Culture and Innovation with support from the National Research Development and Innovation Fund under the Investment into Future call (2020-1.1.6-JÖVŐ) programme.

Authors from the Epidemiology and Surveillance Centre and from the Health Services Management Training Centre work as part of the National Laboratory for Health Security Hungary (RRF-2.3.1-21-2022-00006) supported by the National Research, Development and Innovation Fund.

## Ethical Statement

The study was approved by the Scientific and Ethical Committee of the Medical Council, Hungary. Number of the ethical approval: IV/5698-1/2021/EKU.

## CRediT authorship contribution statement

**Zoltán Vokó:** Writing – review & editing, Methodology, Formal analysis, Conceptualization. **Gergő Túri:** Writing – review & editing, Writing – original draft, Validation. **Beatrix Oroszi:** Writing – review & editing, Validation, Conceptualization. **Éva Belicza:** Writing – review & editing, Data curation. **Tamás Kováts:** Writing – review & editing, Data curation. **Veronika Müller:** Writing – review & editing, Validation, Resources, Investigation. **János Réthelyi:** Writing – review & editing, Validation, Resources, Investigation. **Attila Szijártó:** Writing – review & editing, Validation, Resources, Investigation. **Tamás Masszi:** Writing – review & editing, Validation, Resources, Investigation. **István Takács:** Writing – review & editing, Validation, Resources, Investigation. **János Gál:** Writing – review & editing, Validation, Resources, Investigation. **Zsolt Göböl:** Writing – review & editing, Validation, Resources, Investigation. **Attila Szabó:** Writing – review & editing, Validation, Resources, Investigation. **Csaba Varga:** Writing – review & editing, Validation, Resources, Investigation. **Dávid Becker:** Writing – review & editing, Validation, Resources, Investigation. **Béla Merkely:** Writing – review & editing, Validation, Supervision, Resources, Investigation, Funding acquisition.

## Declaration of competing interest

The authors declare the following financial interests/personal relationships which may be considered as potential competing interests: Bela Merkely reports financial support was provided by National Research Development and Innovation Fund of Hungary. Beatrix Oroszi reports financial support was provided by National Research Development and Innovation Fund of Hungary. If there are other authors, they declare that they have no known competing financial interests or personal relationships that could have appeared to influence the work reported in this paper.

## Data Availability

The authors do not have permission to share data.

## Appendix Supplementary materials

**Supplementary Materials:** Supplementary document 1: ICD-10 codes of diseases in the history of patients. Image, application 1
