# Supplementary material for: Association between COVID-19 vaccination and progression to severe outcomes in hospitalized COVID-19 patients in Hungary during the pre-Omicron era of the COVID-19 pandemic
Source: Virus Res. 2025 Sep 18;361:199633. doi: 10.1016/j.virusres.2025.199633 (PMC12494597; doi:10.1016/j.virusres.2025.199633)
Supplement: Supplementary file 1 — Supplementary Materials: Supplementary document 1: ICD-10 codes of diseases in the history of patients. [file mmc1.docx]

**Supplementary document 1**

ICD-10 codes of diseases in the history of patients:

- cardiovascular disease including ischemic heart disease and heart failure (ICD-10 codes: I09.9, I11.0, I13.0, I13.2, I25.5, I42.0, I42.5-I42.9, I43, I50, P29.0, I20, I21, I22, I23, I24, I25, I21, I22, I25.2)
- cerebrovascular disease (G45, G46, H34.0, I60–I69), type-2 diabetes (E11, E12, E13, E14)
- chronic obstructive pulmonary disease: (I27.8, I27.9, J40, J41, J42, J43, J44, J45, J46, J47, J60, J61, J62, J63, J64, J65, J66, J67, J68.4, J70.1, J70.3)
- malignancies (C00-C26, C30-C34, C37-C41, C43, C45-C58, C60-C80, C97, C81-C85, C88, C96, C90.0, C90.2, C91, C92, C93, C94, C95)
